# The effects of sedentary behaviour on patients with peripheral arterial Disease: A systematic review

**DOI:** 10.1016/j.pmedr.2023.102424

**Published:** 2023-09-18

**Authors:** Marwa Said, Baker Ghoneim, Jennifer Jones, Wael Tawfick

**Affiliations:** a School of Medicine, University of Galway, Ireland; b National Institute for Prevention and Cardiovascular Health, Ireland

**Keywords:** Sedentary time, Sedentary behavior, Peripheral arterial disease, Intermittent claudication, Sitting time

## Abstract

Sedentary behavior has recently emerged as a risk factor for cardiometabolic diseases. The objective of this review was to assess the relationship between sedentary behavior and peripheral arterial disease (PAD). Using the Preferred Reporting Items for Systematic Reviews and Meta-Analysis (PRISMA) guidelines, we performed an electronic search across databases including Cochrane Central Register of Controlled Trials, Embase, MEDLINE (Ovid), CINHAL and PubMed to identify studies focusing on sedentary behavior and PAD. A total of 678 records fulfilled eligibility; 166 duplicates were removed, 487 were excluded at the title and abstract level and 15 studies were excluded at the full article level. Thus, our review comprised 10 studies of 20,064 patients with mean age 67.4 years. The average sedentary time was 544.9 min/day. The current review findings indicate that patients with PAD exhibited prolonged periods of sedentary behavior. Furthermore, sedentary behavior among patients with PAD was associated with lower survival rates. The included studies also reported varied outcomes regarding walking distance with some showing an association between reduced sedentary behavior and increased total walking distance. A randomized controlled trial in this review highlighted that reducing sedentary time among patients with PAD improved walking distance. Therefore, the connection between sedentary behavior and PAD seems to be bidirectional. Sedentary time could contribute to PAD development, and PAD-related symptoms may lead to prolonged sedentary behavior. A call for research investigating the link between PAD and sedentary time. Additionally, intervention studies are needed to target the reduction of sedentary time in patients with PAD.

## Highlights

• A possible bidirectional link between sedentary behavior and peripheral arterial disease (PAD).
• Interrupting prolonged sedentary periods for patients with PAD can yield positive outcomes.
• Triaxial accelerometer placed on hip associated with higher accuracy in sedentary time assessment.
• The sedentary group showed the lowest survival rate among individuals with PAD.
• More studies are needed to understand the relationship between sedentary behavior and PAD.

## 1 Introduction

Worldwide, peripheral arterial disease (PAD) is a major public health challenge affecting more than 200 million patients (Zemaitis et al., 2022). Being an atherosclerotic disease, many risk factors have been identified for PAD including age, smoking, dyslipidemia, hypertension, diabetes and physical inactivity. Sedentary behavior (SB) has been identified as an independent risk factor for cardiovascular diseases (CVDs) including PAD. Sedentary behavior refers to sitting or reclining postures and activities characterised by energy expenditures ≤ 1.5 Metabolic Equivalent of Tasks (METs) (Tremblay et al., 2017).

According to the World Health Organization (WHO), physical activity incorporates any movement performed by skeletal muscles that necessitates energy expenditure. For individuals aged 18–64 years, WHO recommends engaging in either 150–300 min of moderate-intensity aerobic physical activity, 75–150 min of vigorous-intensity aerobic physical activity, or a balanced mix of both throughout the week (WHO, 2022). Whereas physical inactivity is defined as performing an insufficient amount of moderate to vigorous physical activity (Tremblay et al., 2017). Hence, individuals can be physically active according to recommended guidelines while still spending a considerable amount of time in sedentary behavior.

Mechanistically, sedentary behavior links to the development and worsening of PAD (Kulinski et al., 2015; Unkart et al.,2020). High levels of sedentary time are associated with increased inflammation, high blood glucose, and high lipid profiles (Farah et al., 2016a). Accordingly, sedentary behavior has a negative impact on metabolic disorders such as diabetes mellitus, hypertension, and dyslipidaemia, which all or individually and collectively lead to PAD (Park et al., 2020).

Being a chronic disease, PAD has a great impact on lifestyle (Rocha-Neves et al., 2020). Individuals with PAD had an increased 1-year relative risk of adverse events, stroke, heart failure, cardiovascular mortality and all-cause mortality compared to those without PAD (Andersen et al.,2021). Despite its significant impact on morbidity and mortality, PAD has received relatively less research or public attention (Song et al., 2019). The current available literature lacks a standardized definition of sedentary behavior, leading to variations in how it is measured and reported across studies. Also, many studies use cross-sectional designs and limited longitudinal studies tracking individuals over time. Few intervention studies focus on reducing sedentary time among PAD patients.

We hypothesized that increased sedentary behavior is associated with a higher prevalence and severity of PAD among individuals. Therefore, the aim of this review was to evaluate sedentary time among patients with PAD and the effects of prolonged sedentary time on PAD.

## 2 Methods

This systematic review was performed and reported in adherence with the Preferred Reporting Items for Systematic Reviews and Meta-Analyses (PRISMA) guidelines (Page et al., 2021) following the PICOST structure (Population, intervention, Comparator, Outcome). The search was conducted between 20th of March 2022 and 29th of May 2022. We registered the study at PROSPERO International prospective register of systematic reviews (study ID: CRD42023408729).

### 2.1 Ethics approval and consent to participate

Since this is a systematic review, the acquisition of data from human subjects was not necessary, and therefore, ethical approval was not required. Given the nature of this study written informed consent was not considered necessary.

### 2.2 Search strategy

We conducted searches in the following electronic databases: Cochrane Central Register of Controlled Trials (CENTRAL) in the Cochrane library, Embase, MEDLINE (Ovid), CINHAL and PubMed. We utilized Medical Subject Headings (MeSH) descriptors during our search, modifying them accordingly for each database. The terms used included, but were not restricted to “peripheral arterial disease,” “sedentary time,” “sedentary behavior,” “prolonged sitting,” and “intermittent claudication” (Appendix 1). We searched all databases from their inception to May 2022. Search results were downloaded and imported into Rayyan. Rayyan (https://rayyan.ai) is a free web and mobile application designed to help and speed up the initial process of screening and selecting studies (Ouzzani et al., 2016).

## 3 Eligibility criteria

### 3.1 Population

Our review considered studies involving adults (≥18 years) with PAD. Patients were considered to have confirmed PAD if they had any of the following: an ankle-brachial index (ABI) of less than 0.90 in one or both lower extremities, a toe brachial index of less than 0.60, or if arterial occlusive disease was detected in one lower extremity by duplex ultrasonography, computed tomographic angiography, or magnetic resonance angiography (Frank et al., 2019).

### 3.2 Interventions

We included studies looking at interventions to improve sedentary time among patients with PAD, such as behavioral interventions, advice and coaching or remote interventions using computer-based prompting to stand/walk, or software programme incorporates self-monitoring and personal goal setting or wearables.

### 3.3 Comparator

No intervention or minimal intervention. In the context of this review minimal intervention could include online videos containing health recommendations related to PAD or covering topics like general PAD facts.

## 4 Outcome measures

### 4.1 Primary outcome

• Sedentary time in patients with PAD.

### 4.2 Secondary

• Major Adverse Cardiovascular Events (MACE)
• Walking distance.

### 4.3 Study designs

• We included all types of studies that report on sedentary behavior/time and PAD among adults (≥18 years). In our systematic review, we included different study designs such as randomized controlled trial (RCT) design, controlled clinical trials (CCT) and observational studies concentrating on the reporting of sedentary behavior in patients with PAD.

### 4.4 Timing

• The search was performed without restrictions on publication date.

### 4.5 Screening process

We imported titles and abstracts identified from the search strategy into Rayyan (https://www.rayyan.ai). Titles and abstracts of identified studies were screened by two review authors (M.S. and B.G.) to detect their eligibility to be included in the review. Any conflicts were solved by discussion between the two authors. If disagreement persisted, two further authors (J.J. and W.T.) were invited to arbitrate. A similar process was conducted to screen full-text articles.

### 4.6 Data extraction and management

Independently, two review authors (M.S. and B.G.) extracted the data from the incorporated studies. Any disagreement was dealt with by discussion or by asking the other reviewers (J.J. and W.T.). According to general recommendations for dealing with missing data, we reached out to the investigators who had conducted the original research to request the missing data. Data from the selected articles was recorded in a Microsoft Excel spreadsheet by two separate authors (M.S. and B.G.). This data encompassed details about the study, such as the country where it was conducted, the sample size, methods for measuring sedentary time and the main outcomes.

### 4.7 Assessment of bias in conducting the systematic review

Two review authors (M.S. and B.G.) separately evaluated the quality of risk of bias for all included studies using the Newcastle-Ottawa Scale (NOS) (Wells et al., 2014) The NOS assesses studies based on three main criteria: selection of study groups, comparability of groups, and outcome of interest as described in Table 1. Each study is awarded a number of stars, with a higher star count indicating higher methodological quality. In our analysis, the NOS scores ranged from 4 to 6 stars suggesting varying levels of methodological consistency among the included studies.

**Table 1:** The Newcastle- Ottawa Quality Assessment Scale of the included studies.

**Author, year**	**Selection**				**comparability**	**outcome**		**Study quality**
Gerage et al, 2019		*	*	*	-	*	*	Good
Hernandez et al, 2019			*	*	–	*	*	Fair
Whipple et al, 2019			*	*	–	*	*	Fair
Whipple et al, 2020			*	*	–	*	*	Fair
Parson et al, 2016		*	*	*	–	*	*	Good
Laslovich et al, 2020	*		*	*	*	*	*	Good
Delaney et al, 2013	*	*	*		–	*	*	Good
Kulinski et al,2015	*	*	*	*	–	*	*	Good
Gardner et al,2021		*	*	*	–	*	*	Good
Unkart et al, 2020	*	*	*		–	*	*	Good

Table 1: The table presents the Newcastle-Ottawa scale (NOS) assessing risk of bias in 3 domains (selection, comparability, and outcome) with an overall judgment of study quality (poor, fair or good). An increased number of stars can imply decreased bias.

### 4.8 Data synthesis

We had intended to pool multiple studies with similar enough data to perform a *meta*-analysis to identify this common effect. We had planned to use the random-effects model (Higgins et al. 2019). Unfortunately, only two studies focused on interventions aimed at improving sedentary time among patients with PAD. The first study was a randomized controlled trial by Laslovich et al. (2020), while the second study was a pilot study that used a single-group, repeated-measures design without a control group, conducted by Whipple et al. (2020).

## 5 Results

**Fig. 1:**
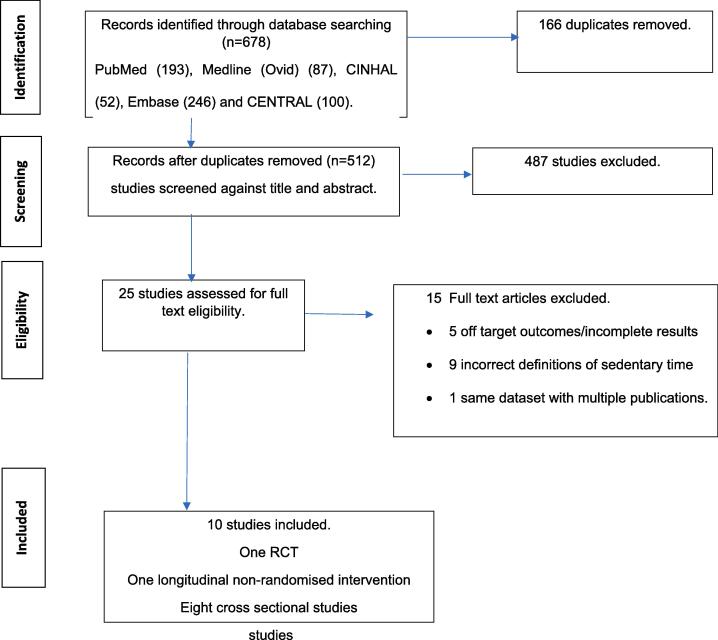
**PRISMA flowchart of the selection process.** A PRISMA flowchart illustrates a total of 512 title and abstract records were screened, out of which 487 records were excluded. Twenty-five studies were assessed for full-text eligibility. A total of 15 reports were excluded, resulting in 10 studies being included in the current systematic review.

The database searches according to our search strategy, resulted in 678 records, of which 166 were removed due to duplication as shown in the PRISMA flowchart (Fig. 1). A total of 512 title and abstract records were screened, out of which 487 records were excluded. Twenty-five studies were assessed for full-text eligibility. A total of 15 reports were excluded, resulting in 10 studies being included in the current systematic review.

### 5.1 Excluded studies

As our primary focus was sedentary time, we excluded 15 reports for various reasons: Mattioli et al. (2018), Gardner et al. (2006) and Lanzi et al. (2021) did not provide information on sedentary time. Ritti-Dias et al. (2022) and Peri-Okonny et al. (2020) reported on the percent of sedentary patients. Germano-Soares et al. (2021) utilized the same patient dataset presented by Gerage et al. (2019) leading to their inclusion as a single study.

Amidou et al. (2018) defined sedentary behavior as < 150 min of moderate-intensity activity per week or equivalent. Berger et al. (2013) classified those engaging in vigorous leisure time exercise at least once a week as active; others were sedentary. Krishnan et al. (2018) categorized the sedentary group as those individuals not meeting the criteria of 30 min/day for 5 days/week of physical activity. Wilson et al., (2011) defined sedentary time to involve behaviors such as sitting, sleeping, or reclining, while also specifying that it excludes any form of lifetime recreational activity (LRA). Some studies incorporated sleep in sedentary time definition. Farah et al. (2016a) and Farah et al. (2016b). McDermott et al. (2011) and McDermott et al. (2016) showed no distinction between reclining during wakefulness, nap time, and sleep time. finally, Gardner et al. (2008) calculated sedentary time by combining sedentary time and light intensity activity. Therefore, neither of these studies fulfilled the criteria for defining sedentary time, which involves sitting/lying behaviors or activities with energy expenditure of 1.5 METs or less (Park et al.,2020).

### 5.2 Included studies

A total of 10 studies were included in this review. They varied in study design and outcome measures. Two studies used interventions to modify sedentary time among patients with PAD. One was a randomized controlled trial (Laslovich et al., 2020), The other was a prospective cohort study (Whipple et al.,2020). The time for the intervention in both was 12 weeks. The remaining eight studies were all cross-sectional in design (Gerage et al., 2019, Hernandez et al., 2019, Whipple et al., 2019, Parsons et al., 2016; Kulinski et al.,2015; Unkart et al.,2020; Gardner et al.,2021; and Delaney et al., 2013). An additional study by Germano-Soares et al. (2021) will also be discussed. Germano-Soares et al. (2021) and Gerage et al. (2019) utilized the same patient dataset, leading to their inclusion as a single study due to multiple publications by the same group. Gerage et al. (2019) was considered the primary study, while both are mentioned for different outcomes.

Detailed descriptions of the included studies’ basic characteristics and summary of results are presented in Table 2. The included studies were published between 2013 and 2022. The total number of individuals included was 20,064 patients. The range of individuals included in the studies covered in this review varied from 10 to 7,609 individuals with an overall mean age of 67.4 years (range 40–96). One study included men only (Parsons et al.,2016) and the remaining nine included both genders, with females representing 39.6%. The current review involved population from a range of countries. One study was conducted in the United Kingdom, one in Brazil, and eight in the United States.

**Table 2:** Descriptive characteristics of studies included in the review with main outcome(s).

**Reference** **Author(s)/year**	**Study design**	**Population**	**Sedentary time measurement**	**Main outcome(s)**
Gerage et al,2019	Cross sectional study.	174 patients (43–96 years) with intermittent claudication (Brazil)	Actigraph GT3X + triaxial accelerometer.	**Sedentary time** Sedentary time was on average 640 ± 121 min/day. **Waking distance:**Using the walking impairment questionnaire (WIQ), the mean (score) walking distance (m) was 22.7 (22.2) .The 6MWT (m) was 326.6 (92.7) .total walking distance was not associated with adherence to PA in PAD patients (OR 1.01 95 %CI (0.99; 1.02) (p > 0.05)).
Hernandez et al., 2019	Cross sectional study.	44 Patients with PAD (USA)	Actigraph GT1M accelerometer	**Sedentary time** Sedentary time was on average 433.45 ± 29.9 min/ day
Whipple et al,2019**(USA)**	A concurrent mixed methods design	Convenient sample of 10 adults aged 65 years and older with PAD and diabetes	Actigraph wGTX3-BT accelerometer	**Sedentary time**Participants spent 66.9% (range 53–78%) of their time in sedentary behavior. **Walking distance:**WIQ mean (SD) was 35.7 (34.7) ranging from 4.3 to 100. The 6MWT (feet) ranging from 480 to 1615 with sedentary participants achieved lower 6MWT distances;77% sedentary was associated with 480 feet and 61% sedentary was associated with 1615 feet 6MWT.
Parsons et al,2016 **(UK)**	Cross sectional .	945 men from the British Regional heart study, mean age 78.4 years. The British Regional Heart Study is a prospective, population-based cohort study following 7735 men recruited from primary care in 24 British towns	Actigrap GT3X accelerometer.	**Sedentary time** Sedentary time was on average 640 ± 84 min/day among low ABI patients. The percentage of time spent sedentary among low ABI patients was 75.9% vs 71.2 % in normal/borderline ABI patients. Each extra 30 min of SB was associated with an OR of 1.19 (95% CI 1.07, 1.33) for a low ABI.
Laslovich et al, 2020 (USA)	RCTIntervention: PA sedentary reduction (PASR) (n = 19).received bimonthly online video series and a 12-wk interactive homebased online sedentary activity reduction programme (GRUVE) . The GRUVE software programme incorporates self-monitoring, personal goal setting, real-time feedback, problem solving, and planning to facilitate increases in daily lifestyle PA and reductions in sedentary behavior.Control: Attention control group (n = 19) received bimonthly online videos involving health recommendations related to PAD (general PAD facts and figures, hypertension, diabetes, cardiovascular disease prevention, tobacco cessation, and nutrition). Participants were asked to continue normal daily activities and routines during the 12-wk study period	38 participants with asymptomatic PAD (APAD)	ActivPAL-3™ Micro activity monitor.	**Sedentary time** Sedentary time was on average in control = 9.46 ± 0.82 h/day vs 9.70 ± 0.68 h/day in intervention group.The intervention group significantly decreased daily sit/lie hours (−0.80 ± 0.87 vs 0.18 ± 0.77P = 0.001). **Walking distance:** Mean ± SD 6MWT (m) was 358.2 ± 89.8 in control group vs intervention 354.5 ± 98.5 at base line. After 12 weeks of intervention the mean 6MWT (m) was 364.6 ± 85 in control vs 467 ± 100.6 in intervention (*P* < 0.001).
Gardner et al,2021 (USA)	Observational study	386 patients were eligible (Fontaine II/Rutherford grade I PAD)	The Johnson space centre (JSC) physical activity scale	**MACE** The sedentary group represented 12.4% During follow up, 66.6% died consisting of 83.3% from the sedentary group, 64.3% from the light-intensity physical activity group, and 64.0% from the moderate- to vigorous-intensity physical activity group.
Kulinski et al., 2015 (USA)	Descriptivedata from the National Health and Nutrition Examination Survey (NHANES)	1443 participants. aged 40 years and older.	ActiGraph accelerometer.	**Sedentary time** Sedentary time was on average 454 ± 144 min/day.Sedentary time was positively associated with a low ABI ((OR) 1.22, (95% CI, 1.03–1.43) ; P = 0.02)
Whipple et al,2020 (USA)	A concurrent mixed methods design Patients completed 2–3 supervised exercise therapy (SET) sessions per week for 12 weeks consisting primarily of repeated bouts of treadmill walking exercise. Sessions broadly followed an established protocol for patients with PAD but were individualized according to patient needs. Exercise intensity wa	44 patients newly enrolled in SET programmes having PAD and DM	A wrist-worn Actigraph wGTX3-BT accelerometer	**Sedentary time** Sedentary time was on average 444.2 ± 101.8 min/day. After 12 weeks of SET participants had a 2.8% increase in the average minutes of sedentary time. Although there was substantial variability, ranging from a 40% decrease to a 38% increase in average minutes of sedentary time per day. There were no statistically significant changes in sedentary activities from baseline to 6 weeks or from baseline to 12 weeks. **Walking distance**6MWT total distance mean (SD) at baseline was 315.5 (94.4) m vs 344.5 (85.1) m after 12 weeks (P = 0.002; (95% CI = 11.4–––46.6)). In addition, improvements in the distance of the WIQ was noted 30.4 (25.7) at baseline vs 38.8 (27.7) after 12 weeks (P = 0.008; (95% CI = 2.3––14.5)).
Unkart et al,2020 (USA)	Observationalthe Hispanic Community Health Study/Study of Latinos (HCHS/SOL)	7,609 eligible Hispanic/Latinos individuals aged 45–74 years old.	Actical accelerometer	**Sedentary time**The median sedentary time was 12.2 (IQR, 11.1–13.3) hr/day. The prevalence of PAD was 5.4%Sedentary time had a significant overall (p = 0.048) association with PAD.Sedentary time was associated with higher odds of PAD, with the highest sedentary time had (OR = 1.49; 95% CI = 1.02,2.18) times higher odds of PAD than patients with the lowest sedentary time.
Delaney et al, 2013	Observational study.	5656 patients (ABI between 0.90 and 1.40) out of the Multi-Ethnic Study ofAtherosclerosis (MESA sample 6,814)	The typical week physical activity survey’	• The incidence of PAD was (n = 161). PAD patients were more sedentary, performed less vigorous and moderate activity.An association between physical activity/ sedentary behavior and the progression to low ABI among the sedentary group unadjusted RR = 1.20, 95 %CI (0.94, 1.52).Greater intentional exercise reduced the risk of incident PAD (RR = 0.85, 95% CI 0.74, 0.98).

Table 2. The table provides a comprehensive overview of the design, population, sedentary time assessment and outcome(s) of the included studies (10 studies). It highlights the specific outcome(s) under investigation, including sedentary time, walking distance, and the presence of Major Adverse Cardiovascular Events (MACE) where applicable.

Studies reporting on the primary outcome could be sub-grouped into categories as follow:

Studies reporting on the association between sedentary time and ABI/PAD.

Studies report on sedentary time (min/day or percentage) among patients with PAD:

Gerage et al., (2019), Hernandez et al., 2019, Whipple et al., 2019, Whipple et al., 2020, Parsons et al., (2016) and Laslovich et al., (2020),

Studies comment on the association between sedentary time and low ABI regardless of symptoms:

Delaney et al., 2013, Whipple et al., 2020, Laslovich et al., (2020), Hernandez et al., (2019), and Gerage et al., (2019).

Studies report on sedentary time and odds of having PAD:

Kulinski et al., (2015), Parsons et al., (2016) and Unkart et al., (2020).

Studies aim at reducing sedentary time among patients with PAD.

Laslovich et al., (2020) and Whipple et al., (2020).

Studies reporting on MACE.

• Gardner et al., (2021).

Studies reporting on walking distance.

Gerage et al., (2019), Laslovich et al., (2020), Whipple et al., (2019) and Whipple et al., (2020).

## 6 Outcomes

### 6.1 Studies reporting on the association between sedentary time and ABI/PAD

**Fig. 2:**
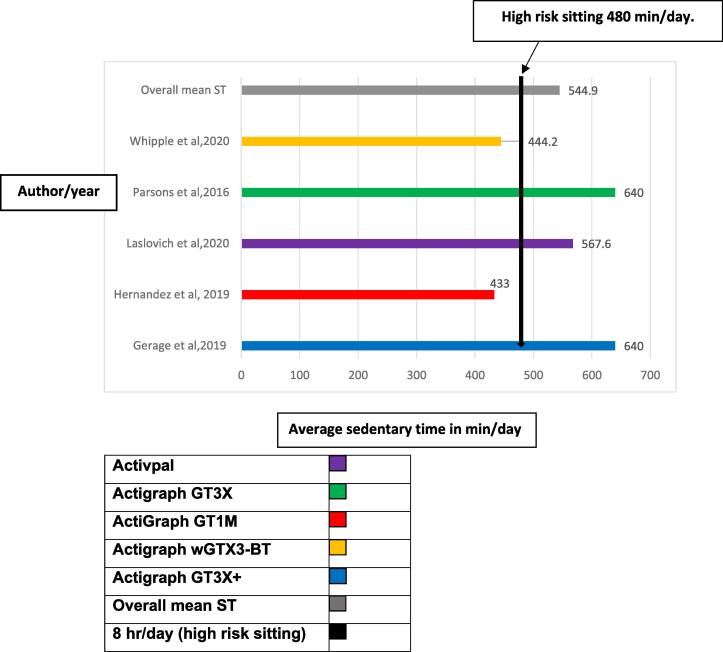
**The mean sedentary time (min/day) among studies using accelerometer for sedentary time assessment.** A bar chart illustrates the various types of accelerometers utilized in the studies that were included in the current review to objectively measure sedentary time in relation to high-risk sitting (480 min/day), with the high-risk sitting being represented by the colour black.

The included studies defined sedentary time as any waking behavior such as sitting or lying with an energy expenditure of 1.5 METs or less (Park et al.,2020). The 10 included studies used various tools, subjective or objective, to measure the sedentary time outcome. Eight studies used motion sensors/accelerometers as objective measurement tools. Across these eight studies, devices varied widely as shown in Fig. 2.

### 6.2 Studies report on sedentary time (min/day or percentage) among patients with PAD

Six studies reported on sedentary time (min/day) in patients with PAD using accelerometer (Gerage et al., 2019, Hernandez et al., 2019, Whipple et al., 2019, Whipple et al., 2020; Parsons et al.,2016; and Laslovich et al.,2020). Whipple et al., (2019) reported on percentage of sedentary time during waking hours. They reported that patients with PAD spent 66.9% (range 53–78%) of their time in sedentary behavior. The remaining five studies reported on mean sedentary time minutes/day. The overall mean sedentary time across these five studies was 544.9 mins/day ranging from 433 to 640 mins/day (Fig. 2).

### 6.3 Studies comment on the association between sedentary time and ABI regardless of symptoms

Five studies reported on sedentary time and ABIs. Delaney et al., (2013) noted an association between sedentary behavior and the progression to low ABI among the sedentary group unadjusted RR = 1.20, 95 %CI (0.94, 1.52). Four studies investigated the association between sedentary time and low ABI: (Whipple et al, 2020), (Laslovich et al, 2020), (Hernandez et al, 2019), and (Gerage et al, 2019). The mean sedentary time across these studies was 521.2 min/day.

High sedentary time (>480 min/day) (Stamatakis et al., 2019, WHO, 2020) was reported in two studies with low ABIs (Laslovich et al, 2020) and (Gerage et al, 2019) with mean sedentary time of 605 min/day. Both used triaxial accelerometers to assess sedentary time.

Whereas moderate sedentary time (240–480 min/day) was reported in two studies with low ABIs (Whipple et al, 2020) and (Hernandez et al, 2019 with mean sedentary time of 438.9 min/day. One study used a triaxial wrist accelerometer (Whipple et al, 2020) while, the other utilized a uniaxial accelerometer (Hernandez et al, 2019).

### 6.4 Studies report on sedentary time and odds of having PAD

Three studies reported on the sedentary time and odds of having PAD/low ABI: Kulinski et al. (2015) Parsons et al. (2016) and Unkart et al. (2020). Kulinski et al. (2015) revealed an odds ratio (OR) of 1.22 (95% CI: 1.03–1.43) for a low ABI with high sedentary time. Similarly, Parsons et al. (2016) showed that each additional 30 min of sedentary time was linked to an OR of 1.19 (95% CI: 1.07, 1.33) for a low ABI. Unkart et al. (2020) indicated an OR of 1.16 (95% CI: 1.02–1.31) for PAD with sedentary time.

### 6.5 Studies aim at reducing sedentary time among patients with PAD

Laslovich et al., (2020) and Whipple et al., (2020) assessed the impact of interventions aiming at modifying sedentary time among patients with PAD. Laslovich et al., conducted an RCT to assess the effect of sedentary time reduction on patients with PAD. The intervention involved a 12-week interactive homebased online sedentary activity reduction programme, which incorporated self-monitoring, personal goal setting, real-time feedback, problem solving, and planning. The intervention significantly decreased daily sit/lie minutes (−48 ± 52 vs 11 ± 46; *P* = 0.001).

Whipple et al., (2020) conducted a pre/post longitudinal prospective study. Patients were asked to complete 2 to 3 Supervised Exercise Therapy (SET) sessions per week for 12 weeks. In contrast to Laslovich et al., the authors of this study reported a 2.8% increase in the average minutes of sedentary time per day from baseline at 12 weeks, following SET. However, there was substantial variability, ranging from a 40% decrease to a 38% increase in average minutes of sedentary time per day.

### 6.6 MACE

One study (Gardner et al.,2021), reported on mortality and sedentary time among patients with PAD. Of the 386 patients included, 257 (66.6%) died during the follow-up period. Survival rate was lowest in the sedentary group. Mortality rate was 83.3% in the sedentary group, 64.3% in the light-intensity physical activity group, and 64.0% in the moderate to vigorous-intensity physical activity group**.**

### 6.7 Walking distance

Four studies reported on walking distance Gerage et al., (2019), Laslovich et al., (2020), Whipple et al., (2020) and Whipple et al., (2019). Two of these studies reported on walking distance, however, did not report on an association with sedentary time. Whipple et al., (2020) and Gerage et al., (2019).

Following a 12-week intervention to reduce sedentary time, Laslovich et al. (2020) noted a significant improvement in walking distance. The mean (SD) 6MWT (m) improved from 354.5 (98.5) to 467 (100.6) in the intervention group, compared to a change from 358.2 (89.8) to 364.6 (85) in the control group (*P* < 0.001).

In a cross-sectional study, Whipple et al., (2019) reported on the association between sedentary time and walking distance. Using the 6MWT (feet) as a measurement of walking distance, persons with greater sedentary time percentage tended to report lower 6MWT distances. Individuals with sedentary time of > 70% had a 6MWT distance of < 1000 feet. Whereas Individuals with sedentary time < 70% had 6MWT distance of > 1000 feet.

Germano-Soares et al., (2021) utilized the same patient dataset as Gerage et al., (2019) and used a compositional *iso*-temporal substitution to detect the effect of reallocating 30 min per week from sedentary to MVPA. This allocation was associated with higher total walking distance (TWD) in men and women. The authors denoted that reducing sedentary time may lead to greater walking distance in patients with PAD.

### 6.8 Key findings from observational and interventional studies in the review

#### 6.8.1 Interventional studies

Two studies, Laslovich et al. (2020) and Whipple et al. (2020) studied interventions modifying sedentary time in patients with PAD. Laslovich et al. conducted an RCT with a 12-week program, significantly reducing sedentary time (−48 ± 52 vs 11 ± 46; *P* = 0.001). Whipple et al. (2020) performed pre/post SET sessions for 12 weeks. Sedentary time varied, with an average 2.8% increase, but wide individual range.

#### 6.8.2 Observational studies

Gerage et al., (2019), Whipple et al., 2019, Hernandez et al., 2019, Parsons et al., (2016), Delaney et al., (2013), Kulinski et al., (2015), Gardner et al., (2021) and Unkart et al., (2020). The research findings suggest that patients with PAD revealed extended periods of sedentary behavior. In terms of walking distance outcomes, study results were variable, with some indicating a link between decreased sedentary behavior and increased total walking distance. Additionally, the sedentary group experienced the lowest survival rate.

#### 6.8.3 Discussion

To our knowledge this is the first systematic review that highlights the association between sedentary time and PAD. The results from the current review from eight observational studies, one cohort and one RCT based on 20,064 persons revealed concerning levels of sedentary time in the PAD population.

The overall mean sedentary time across studies that reported on sedentary time in min/day was 544.9 mins/day ranging from 433 to 640 mins/day. Sedentary behavior among patients with PAD was associated with lower survival rates. Some studies showed a relationship between reduced sedentary behavior and increased overall walking distance. Additional randomized controlled trials are needed to explore the effects of reducing sedentary time in patients with PAD. These trials should also evaluate the feasibility and acceptability of different intervention approaches.

#### 6.8.4 Methods to assess sedentary time

Numerous subjective tools, such as questionnaires, diaries and logs have been used to assess sedentary time (Bakker et al., 2020). Subjective methods, such as questionnaires, are subject to measurement error and response bias (Aunger and Wagnild, 2022). Objective tools, such as accelerometers have been used increasingly in research, providing a more accurate measurement of sedentary time by capturing all types of sedentary behavior (Atkin et al., 2012). The triaxial accelerometers assess sedentary time better, as it measures movements in the three dimensions of space, while the uniaxial accelerometer measures only one dimension, so it may lack some movements (Plasqui et al.,2005); (Vanhelst et al.,2012).

In this review, Hernandez et al., (2019) reported a mean sedentary time of 433 min/day which is less than the 480 min/day cut off for high-risk sedentary time (Stamatakis et al., 2019, WHO, 2020). There is a possibility they may have underestimated the sedentary time, as they used a uniaxial accelerometer (ActiGraph GT1M) waistband.

The location of accelerometer placement may also influence the accuracy of sedentary time measurement. In a study by Marcotte et al (2020) placed triaxial accelerometers on the right hip and nondominant wrist. Results showed that the wrist underestimated sedentary time. A systematic review noted that placing the device on the hip was associated with higher accuracy compared to the wrist (Lynch et al., 2019). One of the included studies in our review (Whipple et al., 2020) reported a mean sedentary time of less than 480 min/day in patients with PAD, thus not reaching the cut off of high-risk sedentary time (Stamatakis et al., 2019, WHO, 2020). There is a potential that the authors may have underestimated sedentary time measurement, as they used a wrist worn triaxial accelerometer. Thus, triaxial accelerometer placed on hip associated with higher sedentary time assessment accuracy.

#### 6.8.5 Exploring the complex relationship between sedentary time and PAD

The mechanism clarifying the pathogenesis between sedentary time and PAD is still unclear. Many possible explanations have been theorised by existing evidence. Excess sedentary time has independent effects on cardiometabolic biomarkers, such as lipids, glucose metabolism and the vascular system, resulting in atherogenesis (Young et al., 2016). Additionally, time spent in sedentary behavior has been associated with high-sensitive C-reactive protein (hs-CRP), glucose, plasminogen activator inhibitor-1 activity and fibrinogen. Even after adjusting for variables like sex, age, physical activity status, body mass index, and PAD severity, a relationship between sedentary behavior and markers remained evident (Farah et al., 2016b). Another probable mechanism is that sedentary time increases reactive oxygen species, which is associated with the increased cytokine production and other inflammatory markers, eventually leading to endothelial dysfunction (Bianchi and Ribisl, 2015).

Park et al., (2020) noted that sedentary behaviour reduces lipoprotein lipase activity, muscle glucose, protein transporter activities, impairs lipid metabolism, and diminishes carbohydrate metabolism. As a result, patients with more sedentary time have a higher prevalence of diabetes mellitus (DM), a higher body mass index, metabolic syndrome, and obesity than patients with less sedentary time (Farah et al., 2016a). Currently, there is no conclusive evidence indicating a direct contribution of sedentary time to PAD. It's possible that the relationship is more indirect, involving PAD risk factors like DM and obesity.

Evidence from the cross-sectional observational studies by Parsons et al., 2016, Unkart et al., 2020 and Kulinski et al., (2015) revealed that sedentary time was associated with higher odds of having PAD which persisted after adjusting for other traditional PAD risk factors as dyslipidemia, hypertension, and diabetes. Additionally, Unkart et al., (2020) noted that adjustment for hypertension and diabetes minimally reduced the association between PAD and sedentary time. The authors suggested that blood pressure and glucose regulation did not fully mediate the association. Thus, prolonged sedentary time may have other independent damaging effects on the vascular endothelium (Unkart et al., 2020).

From a different perspective, Intermittent claudication, a primary PAD symptom, diminishes walking capacity. Moreover, PAD decreases exercise capacity, leading to sedentary behavior even without leg symptoms (Hamburg and Balady, 2011). Hence, A potential bidirectional relationship might exist; sedentary time could induce inflammatory markers, contributing to atherosclerosis, while PAD-related symptoms could promote increased inflammation, influencing sedentary behavior in turn.

#### 6.8.6 Sedentary time discrepancy: PAD patients vs. Non-PAD individuals

Fullwood et al., 2019 revealed that older adults with PAD had significantly higher total accumulated time spent in sedentary behavior than those without PAD (13.1 min per day, p < 0.02). The increased sedentary time observed in PAD patients could be attributed to many factors, such as reduced mobility, discomfort linked to mobility, and potential limitations in engaging in physical activities. In addition, prolonged periods of sitting have been linked to impaired blood circulation, which is particularly relevant to PAD patients due to their compromised blood flow to the extremities. This heightened sedentary time might contribute to the exacerbation of PAD-related symptoms and further hinder their overall quality of life.

#### 6.8.7 The effect of reducing sedentary time on PAD

The World Health Organization (WHO) emphasizes the importance of reducing sedentary time (WHO,2020). There was only one RCT included in our review (Laslovich et al., 2020). The authors noted that reducing daily sedentary time improved walking distance in the intervention group compared to the control group. In contrast to the above studies, Whipple et al. (2020) performed found no significant changes in any of the sedentary time or physical activity variables from baseline. However, there was substantial variability with some persons experiencing 40% less sedentary time at 12 weeks compared to baseline, whereas others experienced an increase in sedentary time of up to 38% more at 12 weeks than at baseline. These results suggest that reducing sedentary behavior could potentially enhance walking ability and overall physical functioning in patients with PAD. Additionally, focusing on reducing sedentary behavior might be more feasible than encouraging individuals with PAD to consistently engage in regular exercise.

#### 6.8.8 Sedentary lifestyle and all-cause mortality

A *meta*-analysis on sedentary behavior, all-cause, and CVD mortality, reported that a threshold of 6–8 h/day of total sitting increased the risk for all-cause mortality (Patterson et al., 2018). Furthermore, Stamatakis et al., (2019) conducted a large-scale prospective study with 8.9 years of median follow-up for all-cause mortality. The authors revealed that replacing sitting with standing was associated with a small reduction in all-cause mortality risk in low sitters only. Additionally, replacing sitting with walking and vigorous physical activity was associated with a reduction in all-cause mortality risk in high sitters. Therefore, adults should be encouraged to sit less during the day to reduce their daily total sedentary time (Chau et al., 2015); (Stamatakis et al., 2019).

In this review, only one study reported on and mortality among patients with PAD (Gardner et al., 2021). During follow up of this observational study 66.6% of patients with PAD died. There was a significantly higher incidence of mortality in the sedentary group (83.3%) compared to the light-intensity group (64.3%) and the moderate to vigorous-intensity group (64.0%). Similar studies among patients with medical conditions other than PAD have been done and revealed similar associations between a sedentary lifestyle and all-cause mortality (Ku et al.,2018).

## 7 ABI and walking distance

The ABI, a reliable prognostic marker for PAD, and walking capacity, a core clinical measure linked to PAD, relate to endothelial function, inflammation, and various clinical indicators (Gerage et al., 2019). Previous studies revealed that in patients with PAD, lower ABI has been associated with lower walking capacity (Amoh-Tonto et al., 2009); (Brewer et al., 2007). However, Laslovich et al. (2020) found no significant associations between ABI and 6MWT distance. One possible explanation could be that the authors incorporated PAD patients who were asymptomatic.

### 7.1 Physical activity, exercise and PAD

Supervised exercise training is vital for PAD patients, improving function and life quality. However, availability restricts their broader public health use. Thus, promoting increased physical activity remains key in clinical practice for patients with PAD (Gerage et al., 2019). Germano-Soares et al., (2021) noted that modifying 30 min/week from sedentary behavior to MVPA was associated with a higher total walking distance in patients with PAD. These quantified relationships provide valuable insights into the potential benefits of increased physical activity and reduced sedentary time in the context of PAD.

## 8 Conclusion

The current systematic review uncovered the association between sedentary behavior (SB) and peripheral arterial disease (PAD). The link between both conditions was mostly independent to physical activity and appears to be bidirectional. Sedentary time might foster inflammation, contributing to atherosclerosis, while PAD symptoms could restrict mobility, inducing sedentary behavior. Furthermore, sedentary behavior among patients with PAD was associated with lower survival rates. Some studies revealed a link between less sedentary behavior and greater total walking distance. The study suggests implementing large-scale interventions to manage PAD and decrease sedentary behavior, which could lead to significant health benefits and positive outcomes for patients, including reducing the risk of co-morbidities, mortality, and long-term conditions.

**Authors' contribution**

MS, WT, JJ designed the study. MS, WT, JJ, BG assisted in collecting and screening the data. MS, WT, JJ, BG interpreted the data. MS, WT, JJ drafted the initial manuscript. WT, JJ Conducted a thorough and analytical assessment of the manuscript and made necessary improvements and modifications. The final version of the manuscript was authorised for publication by all the authors.

**Disclosure of funding and conflicts of interest**

The author(s) have no conflicts of interest to declare. We acknowledge the University of Galway for covering the publication costs of this work."

## Declaration of Competing Interest

The authors declare that they have no known competing financial interests or personal relationships that could have appeared to influence the work reported in this paper. We sincerely thank the University of Galway for covering the publication fees, which enabled us to share this work more widely

## Figures and Tables

**Table:** 

Peripheral Arterial Disease*
Peripheral Artery Disease*
PAD
Peripheral vascular disease*
PVD
Intermittent Claudication
Claudi*
Ankle brachial index
ABI
Ankle brachial pressure index
ABPI
Critical limb ischemia
Critical limb ischaemia
CLI
Rest pain
Ulcer*
1 OR 2OR 3 OR 4 OR 5 OR 6 OR 7 OR8 OR9 OR10 OR11 OR12 OR13 OR14 OR15 OR16
Sedentary
Sitting
Reclin*
Lying
Inactiv*
Activ*
Behavior*
Behavior*
Tim*
Intervention*
Prolonged
Uninterrupted
Un-interrupted
Physical*
18 AND 24
18 AND 25
18 AND 26
18 AND 27
19 AND 24
19 AND 25
19 AND 26
28 AND 19
29 AND 19
30 AND 19
31 AND 22
31 AND 23
28 AND 20
29 AND 20
30 AND 20
28 AND 21
29 AND 21
30 AND 21
28 AND 22
29 AND 22
30 AND 22
18 OR 32 OR 33 OR 34 OR 35 OR 36 OR 37 OR 38 OR 39 OR 40 OR 41 OR 42 OR 43 OR 44 OR 45 OR 46 OR 47 OR 48 OR 49 OR 50 OR 51 OR 52
17 AND 53

## Data Availability

Data will be made available on request.

## Appendix 1

Search strategy
